# The neuronal migration hypothesis of dyslexia: A critical evaluation 30 years on

**DOI:** 10.1111/ejn.14149

**Published:** 2018-10-06

**Authors:** Luiz G. Guidi, Antonio Velayos‐Baeza, Isabel Martinez‐Garay, Anthony P. Monaco, Silvia Paracchini, Dorothy V. M. Bishop, Zoltán Molnár

**Affiliations:** ^1^ Department of Physiology, Anatomy, and Genetics University of Oxford Oxford UK; ^2^ Wellcome Centre for Human Genetics University of Oxford Oxford UK; ^3^ Division of Neuroscience School of Biosciences Cardiff University Cardiff UK; ^4^ Office of the President Tufts University Medford Massachusetts; ^5^ School of Medicine University of St Andrews St Andrews UK; ^6^ Department of Experimental Psychology University of Oxford Oxford UK

**Keywords:** dyslexia, gene function, neuronal migration, neuropathology, RNA interference

## Abstract

The capacity for language is one of the key features underlying the complexity of human cognition and its evolution. However, little is known about the neurobiological mechanisms that mediate normal or impaired linguistic ability. For developmental dyslexia, early postmortem studies conducted in the 1980s linked the disorder to subtle defects in the migration of neurons in the developing neocortex. These early studies were reinforced by human genetic analyses that identified dyslexia susceptibility genes and subsequent evidence of their involvement in neuronal migration. In this review, we examine recent experimental evidence that does not support the link between dyslexia and neuronal migration. We critically evaluate gene function studies conducted in rodent models and draw attention to the lack of robust evidence from histopathological and imaging studies in humans. Our review suggests that the neuronal migration hypothesis of dyslexia should be reconsidered, and the neurobiological basis of dyslexia should be approached with a fresh start.

**Abbreviations**
ADHD: attention deficit hyperactivity disorder
BA: Brodmann area
CNV: copy number variations
DLD: developmental language disorder
GFP: green fluorescent protein
GWAS: genome‐wide association studies
KO: knockout
MRI: magnetic resonance imaging
mRNA: messenger RNA
NGS: next generation sequencing
PVNH: periventricular nodular heterotopia
RNAi: RNA interference
shRNA: small hairpin RNA

## 1 INTRODUCTION

One of the greatest challenges in understanding susceptibility to neurodevelopmental disorders lies in establishing a connection between studies on human brains, with neuroimaging or neuropathology, and findings at the molecular and cellular levels from studies of gene function in animal or cell models. There is complementarity in the level of granularity each approach can take: while the former typically offers large‐scale features such as gray matter volume, white‐matter tract density and so on, the latter interrogates much more fine‐grained problems such as molecular interactions, formation of synapses or physiological activity. The link between cortical migration defects and neurological and cognitive conditions is well established (Ayala, Shu, & Tsai, 2007; Rakic, 1988; Walsh & Goffinet, 2000). Our review specifically examines the link for dyslexia.

For developmental dyslexia, there was a remarkable convergence of evidence from human studies and functional genetics in the mid‐2000s. This line of work was initiated by a series of postmortem studies on the brain of dyslexic individuals that identified a large number of micro‐abnormalities in the organisation of cortical neurons in key regions of the language network (Galaburda, 1985; Galaburda & Kemper, 1979; Humphreys, Kaufmann, & Galaburda, 1990; Kaufmann & Galaburda, 1989). This led to suggestions that impaired neuronal migration may be a cellular antecedent to dyslexia (Galaburda, 1985, 1992, 1993). With the identification of the first susceptibility genes for dyslexia in the early 2000s, researchers attempting to uncover their function in the brain found that they were involved in precisely this process during cortical development (Meng et al., 2005; Paracchini et al., 2006; Wang et al., 2006). This striking convergence led to the establishment of the hypothesis that dyslexia is a disorder of neuronal migration (Galaburda, LoTurco, Ramus, Fitch, & Rosen, 2006; Paracchini, Scerri, & Monaco, 2007). Specifically, the claim is that newborn neurons derived from the ventricular zone of the cortex fail to move upwards as normal towards the cortical plate and end up misplaced, leading to subtle abnormalities in brain structure, connectivity and, ultimately, function. This view fits with the ideas that (a) most of language and reading processing takes place in the neocortex and (b) that defects leading to problems in these functions must be in place from an early stage in development.

From its origins in the late 1970s, the proposal has achieved a consensus‐like status within much of the research community on language neurobiology. However, with technological advances and new evidence being uncovered, particularly in molecular and functional genetics, the time is ripe for an evaluation of the evidence surrounding the association between neuronal migration and dyslexia.

In this review, we start by outlining the original findings from studies in both humans and animal models that lead to formulate the neuronal migration hypothesis. We then review recent studies on gene function and note concerns over reproducibility of some of those original findings, followed by an evaluation of how the candidate genes studied so far fit into the growing understanding of the genetic architecture of dyslexia. In the light of methodological issues surrounding the neuroanatomical analyses of dyslexia in human histological and imaging studies, the picture that emerges is that evidence for the neuronal migration hypothesis from human studies and animal models is not very robust, suffering from a number of limitations which cast doubt on the original hypothesis. The conclusion is that the link between dyslexia and neuronal migration should be considered with caution.

## 2 DEVELOPMENTAL DYSLEXIA

Developmental dyslexia refers to a deficit in reading ability in individuals with normal intelligence and educational opportunity, and no major sensory abnormalities (World Health Organisation, 2008). It is one of the most common neurodevelopmental disabilities, affecting 5%–12% of school‐aged children across different countries (Peterson & Pennington, 2015). Children with dyslexia are slow to learn to read and, even if they attain adequate reading accuracy, they do not read fluently (Lefly & Pennington, 1991). Dyslexia appears to be a complex, multi‐factorial disorder with a strong genetic component in its aetiology, with heritability estimates from twin studies at 40%–70% (Paracchini et al., 2007). Like other neurodevelopmental disorders, it commonly co‐occurs with conditions including developmental language disorder (DLD; Newbury et al., 2011; Snowling, 2000), attention deficit hyperactivity disorder (ADHD; Germano, Gagliano, & Curatolo, 2010; Gilger, 1992), and mathematical disability (Davis et al., 2014; Ritchie & Bates, 2013), among others (Eicher et al., 2015; Pennington, 2006; Richardson & Ross, 2000).

Despite extensive investigation, the neuropsychological mechanisms underlying dyslexia are not well understood, and proposals range from deficits specific to the phonological system and subtle problems in sensory perception, to impaired attention and motor deficits (for general reviews, see Shaywitz & Shaywitz, 2008; Ramus & Ahissar, 2012; Goswami, 2015; Peterson & Pennington, 2015; Paracchini, Diaz, & Stein, 2016). Although the phonological deficit theory is the most widely accepted, the specific nature of the deficit is a matter of much debate, as proposals typically only account for a subset of the observed abnormalities—a fact further complicated by a lack of consensus in diagnostic criteria and the highly heterogeneous nature of the disorder (Bishop, 2015; Newbury, Monaco, & Paracchini, 2014).

The neural architecture that supports reading involves a complex circuitry largely dependent on the core language network, a left‐lateralised system involving temporo‐parietal areas connected to the inferior frontal cortex via the arcuate fasciculus (Carreiras, Armstrong, Perea, & Frost, 2014; Dehaene, 2009; Friederici & Gierhan, 2013; Hagoort, 2014; Price, 2012). An area of the left fusiform gyrus known as the visual word‐form area responsible for word recognition is generally considered as an important part of the reading circuitry (Dehaene & Cohen, 2011; Logothetis & Sheinberg, 1996), although this cortical region appears to serve functions that go beyond reading (Cohen et al., 2002; Vogel, Petersen, & Schlaggar, 2014). The reading network consists of two major pathways: a dorsal circuit involving the occipital, supramarginal and angular gyri, which connect to the premotor cortex and pars opercularis around Broca's area in the inferior frontal cortex; and a ventral pathway which connects the left fusiform gyrus and the middle/anterior temporal gyrus with the pars triangularis in the frontal cortex (Carreiras et al., 2014; Dehaene, 2009).

A vast number of neuroimaging studies has been conducted over the past couple of decades with the goal of identifying the neurobiological basis of dyslexia. These have identified several features that are commonly observed in cohorts with reading impairment. These studies have predominantly focused on the neocortex and, at the structural level, suggest there is an altered degree of asymmetry in the planum temporale (Altarelli et al., 2014; Bloom, Garcia‐Barrera, Miller, Miller, & Hynd, 2013; Eckert, 2004; Guadalupe et al., 2015), abnormal white matter integrity along the left arcuate fasciulus (Vandermosten, Boets, Wouters, & Ghesquiere, 2012; Zhao, Thiebaut de Schotten, Altarelli, Dubois, & Ramus, 2016), and altered cortical thickness in the visual word‐form area (Altarelli et al., 2013; Monzalvo, Fluss, Billard, Dehaene, & Dehaene‐Lambertz, 2012; Richardson, Seghier, Leff, Thomas, & Price, 2011), among other findings. Functionally, hypoactivation of the left occipitotemporal region is one of the most consistent findings, particularly in the visual word‐form area (Maisog, Einbinder, Flowers, Turkeltaub, & Eden, 2008; Norton, Beach, & Gabrieli, 2015; Richlan, 2012). There are also reports of alterations in regions other than the cortex such as the thalamus (Diaz, Hintz, Kiebel, & von Kriegstein, 2012; Livingstone, Rosen, Drislane, & Galaburda, 1991), the auditory brainstem (Hornickel & Kraus, 2013) and the cerebellum (Stein, 2001). However, there is little consensus with respect to our understanding of the neurobiology of dyslexia (see Norton et al., 2015; Shaywitz & Shaywitz, 2008, for reviews).

Elucidating the genetics of dyslexia has the potential to shed light on to the underlying neuropsychology and neurobiology. Several dyslexia susceptibility *loci* and candidate genes have been identified over the last two decades, with *DYX1C1*,* DCDC2, KIAA0319* and *ROBO1* established as the main candidates from linkage and fine‐mapping association studies (for reviews, see Carrion‐Castillo, Franke, & Fisher, 2013; Kere, 2014; Paracchini et al., 2016). Although these arguably remain as the strongest candidate genes to date, they have not been consistently replicated across studies (e.g., Becker et al., 2014; Carrion‐Castillo et al., 2017; see also Carrion‐Castillo et al., 2013; Scerri & Schulte‐Korne, 2010; for reviews) and they have received little or no support from genome‐wide association studies (GWASs) conducted so far (Eicher et al., 2013; Field et al., 2013; Gialluisi et al., 2014; Luciano et al., 2013; Paracchini et al., 2016). However, it is worth noting that GWAS for dyslexia have been under‐powered so far and variants with the strongest association to dyslexia (e.g., in genes *RBFOX2, ABCC13, ZNF385D, COL4A2* and *FGF18)* failed to survive genome‐wide statistical scrutiny (Eicher et al., 2013; Field et al., 2013; Gialluisi et al., 2014; Luciano et al., 2013). Furthermore, the largest GWASs to date have interrogated association to reading abilities in the normal range of variation as observed in general population samples, rather than investigating a cohort of dyslexics (Newbury et al., 2014; Paracchini, 2011). GWAS have found suggestive evidence for *DCDC2* in dyslexia susceptibility in a study that investigated the genetics of mathematical and reading disability (Davis et al., 2014). In addition, *KIAA0319* was listed in the top 300 genes reported to be significantly associated with general cognitive ability in gene‐based analyses conducted for a large sample (*N* = 280, 360; Davies et al., 2017). A number of other genes and types of variants such as copy number variations (Pagnamenta et al., 2010; Poelmans et al., 2009; Veerappa, Saldanha, Padakannaya, & Ramachandra, 2013) and rare coding mutations in *CCDC136/FLNC, NCAN* and *CEP63* in isolated families (Adams et al., 2017; Einarsdottir et al., 2015, 2017) have been implicated in dyslexia. For recent detailed reviews on the genetics of dyslexia and language disorders, see (Carrion‐Castillo et al., 2013; Graham & Fisher, 2015; Kere, 2014; Newbury et al., 2014; Paracchini et al., 2016).

## 3 HUMAN NEUROANATOMY

**Figure 1:**
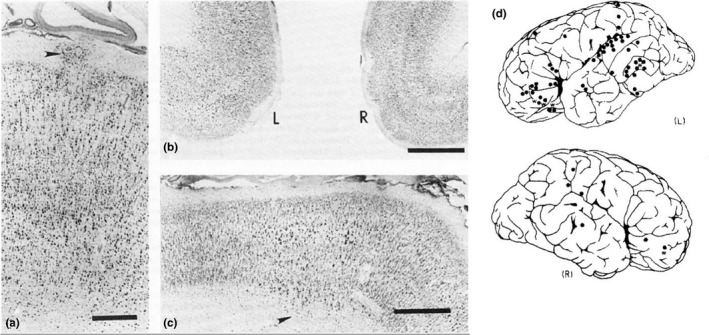
Micro‐abnormalities in the cerebral cortex in postmortem histopathological studies of dyslexia. (a–c) Nissl staining of serial sections from the dyslexia case in Galaburda and Kemper (1979) where the cerebral cortex shows signs of cortical defects in the form of layer 1 ectopias (a, arrowhead; scale = 1 mm), dysplasia in the left cingulate cortex (b; scale = 2 mm), as well as neurons in the white matter (arrowhead) and dyslamination in the occipital cortex (c; scale = 2 mm). (d) The distribution of micro‐abnormalities in a case from Galaburda et al. (1985) showing these to be concentrated around the left peri‐sylvian area of the brain as shown in the schematic diagram of the left (L) and right (R) hemisphere where black dots denote the location of identified defects. Images adapted from Galaburda and Kemper (1979) and Galaburda et al. (1985)

Before the advent of neuroimaging studies, one of the first investigations into the neuroanatomical basis of dyslexia came from the postmortem examination of the brain of a 12‐year‐old boy, which identified abnormalities in the convolutional pattern of the parietal lobes bilaterally, thinning of the corpus callosum and misplaced neurons (i.e., ectopias) in the white matter (Drake, 1968). But it took over 10 years for new evidence to be uncovered, when a team led by Albert Galaburda at Harvard Medical School examined the brains of individuals with dyslexia across three separate reports (Galaburda, 1985; Galaburda & Kemper, 1979; Humphreys et al., 1990). Using histopathological analyses with neuronal and myelin stainings, these studies investigated the brains of eight people, five males and three females, using serial sections spanning the rostro‐caudal length of each brain to capture a detailed picture of their micro‐structure. One of the most prominent findings in these studies was the high incidence of small cortical malformations, typically appearing as layer I neuronal ectopias, with some laminar dysplasia and focal microgyria (Figure 1). The number of anomalies observed in each of the brains varied between 30 and 140, and clustered around the left peri‐sylvian region in the superior temporal gyrus and Heschl's gyrus (Brodmann areas [BAs] 22, 41 and 42), key regions of the language network. As the authors point out, these micro abnormalities resembled somewhat the defects seen in cases published earlier (Cohen, Campbell, & Yaghmai, 1989; Drake, 1968; Levine, Hier, & Calvanio, 1981).

A separate study was conducted to examine whether similar cortical dysgeneses were present in non‐dyslexic brains (Kaufmann & Galaburda, 1989). Previous reports in control samples had identified up to 26 *foci* of anomalous micro‐structure in the neocortex (Morel & Wildi, 1952; Schulze & Braak, 1978) but concerns had been raised over whether these samples were representative of unaffected brains (Kaufmann & Galaburda, 1989). Using their rigorous serial section method, they found that abnormalities similar to those observed in dyslexic brains were present in three out of the 10 non‐dyslexic samples investigated, but appeared in significantly smaller numbers (1–2 per brain) and located away from language‐related areas, predominantly in the cingulate cortex (Kaufmann & Galaburda, 1989). Given focal ectopias and microgyria are characteristic of abnormal neuronal migration during the development of the neocortex, these observations led to initial suggestions that impaired neuronal migration may be a cellular antecedent to dyslexia (Galaburda, 1989, 1992, 1993; Galaburda, Sherman, Rosen, Aboitiz, & Geschwind, 1985). Around the same time, studies of mice with autoimmune disorders exhibiting similar cortical ectopias were found to suffer from auditory deficits similar to those described in dyslexics (Galaburda, 1992; Sherman, Galaburda, Behan, & Rosen, 1987; Sherman, Galaburda, & Geschwind, 1985). Combined with reports of higher incidence of immune deficiencies in the dyslexic population, this provided some added support for the proposal (Galaburda, 1992, 1993; Habib, 2000).

Further evidence in support of this view did not emerge until years later with the use of in vivo human neuroimaging methods. In two consecutive studies, structural magnetic resonance imaging (MRI) was used alongside behavioural tests on adults displaying a type of cortical migration malformation called periventricular nodular heterotopia (PVNH; Chang et al., 2005, 2007), where masses of neurons accumulate near the lateral ventricles bordering the cortical wall. These authors reported that patients with PVNH performed poorly on reading tasks, with their performance resembling that seen in dyslexia. We discuss this evidence further below.

## 4 FUNCTIONAL GENETICS AND NEURONAL MIGRATION

Molecular genetics studies gave further strength and support to the neuronal migration hypothesis. In the early and mid‐2000s, the first candidate genes for dyslexia started to emerge and revealed *DYX1C1*,* DCDC2*,* KIAA0319* and *ROBO1* as the main dyslexia susceptibility genes (for a contemporary review, see Fisher & Francks, 2006; Paracchini et al., 2007). At the time, little was known about the function of these genes inside cells and as part of neural circuits. Questions therefore emerged about their role in the healthy brain and in dyslexia.

*DCDC2* was the first target of functional studies. The *DCDC2* gene encodes a protein containing two doublecortin domains, motifs which had been strongly associated to neuronal migration via similarity (homology) to the *DCX* gene in studies with both humans and rats (Bai et al., 2003; Gleeson et al., 1998). In the light of evidence from human postmortem studies in individuals with dyslexia mentioned above, this suggested that DCDC2 may also be involved in mediating the migration of cortical neurons. Meng et al. (2005) tested this possibility using state‐of‐the‐art methodology called *RNA interference* (RNAi; Davidson & Boudreau, 2007; Rana, 2007) in the developing cortex of rats as a model to probe the function of the protein. This method uses in utero electroporation to deliver DNA constructs to newborn neurons occupying the ventricular wall, at a time when neurons start their migration to the cortical plate (LoTurco, Manent, & Sidiqi, 2009; Reiner, Gorelik, & Greenman, 2012). In this and other similar studies, the DNA constructs encode a *small hairpin RNA* (shRNA) which, when expressed and processed, reduces the production of the protein encoded by the target gene by mediating the degradation of the relevant messenger RNA (mRNA). These shRNA constructs are electroporated into cells together with constructs encoding green fluorescent protein (GFP) so that cells that have and have not been targeted can be identified and distinguished from each other. As a control, the same procedure is conducted in another animal using the same conditions, but using an shRNA construct that has no predicted target (i.e., it should not “interfere” with any gene). The position of neurons transfected in both conditions can then be compared to assess whether the shRNA targeting a specific gene affects neuronal migration. This method offers a fast and inexpensive way to lower or “knock‐down” the activity of a protein by reducing its availability in a given cell or tissue.

**Figure 2:**
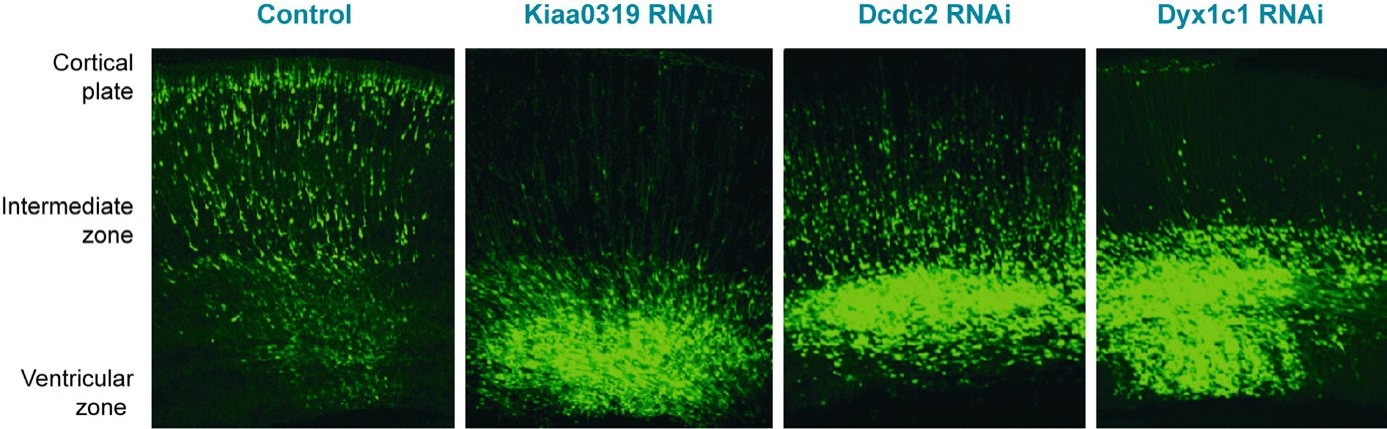
RNA interference against key dyslexia susceptibility genes (*Kiaa0319, Dcdc2, Dyx1c1*) impairs neuronal migration after in utero electroporation in the developing cortex of rat embryos. Images show sections of the developing rat neocortex 4 days after electroporation, with targeted neurons in green due to the presence of green fluorescent protein (GFP) for labelling. In the control experiment, neurons are seen occupying the entire length of the cortical wall, with most neurons in the cortical plate or intermediate zone. A dramatic difference is seen in the case of neurons targeted with small hairpin RNA (shRNA) constructs against *Kiaa0319, Dcdc2* or *Dyx1c1,* as the majority occupy the ventricular or intermediate zone, with only a small proportion in the cortical plate. Adapted from Paracchini et al. (2007)

By delivering *DCDC2*‐shRNA constructs to early neurons in the rat cortex, Meng et al. (2005) knocked down the levels of the DCDC2 protein in neurons at the time they were undergoing migration. If the protein plays an important role in this process, it would be expected that its reduced availability in certain neurons would affect the neuron's ability to move and, as a result, it would fail to occupy its intended position in the cortical plate. As such, in the test condition, it would be expected that the overall distribution of neurons along the cortical plate would be different when compared with control experiments. Four days after transfection, GFP‐expressing neurons in control animals were found predominantly in the cortical plate, whereas in the cortices of embryos transfected with *DCDC2*‐shRNA, the bulk of electroporated cells were significantly further from it, clustering around the intermediate and subventricular zones (Figure 2). This indicates that shRNA knockdown of DCDC2 led to alterations in the migration of neurons in the developing cortex. This finding paved the way for work with the two other main candidate genes: work on *KIAA0319* from our laboratory (Paracchini et al., 2006) and on *DYX1C1* (Wang et al., 2006) revealed a similar effect after knockdown of these genes, suggesting a role for the two proteins in neuronal migration (Figure 2; Gabel, Gibson, Gruen, & LoTurco, 2010; Paracchini et al., 2007).

Several other studies followed in attempts to refine the specific characteristics of the migration defects observed and the cellular events affected by shRNA‐knockdown of each of these genes (Adler et al., 2013; Burbridge et al., 2008; Currier, Etchegaray, Haight, Galaburda, & Rosen, 2011; Peschansky et al., 2010; Rosen et al., 2007). Others also investigated how altered levels of the proteins affected rodent behaviour (Szalkowski et al., 2011, 2012, 2013; Threlkeld et al., 2007). More recently, a gene similar to *KIAA0319* and the only other member of the gene family, *KIAA0319‐Like* (or *KIAA0319L),* also reported to be associated with dyslexia (Couto et al., 2008), was probed for potential links to neuronal migration, and shRNA knockdown experiments also elicited problems in neuronal migration in the form of PVNH (Platt et al., 2013). Furthermore, the other main dyslexia candidate gene, *ROBO1*, was shown to be implicated in cell migration and axon growth, another developmental process that can lead to altered brain connectivity (Hannula‐Jouppi et al., 2005; Lopez‐Bendito et al., 2007; Yuan et al., 1999). A summary of the studies targeting neuronal migration is shown in Table 1. Overall, these results presented a remarkable overlap in function between the main dyslexia susceptibility candidate genes which closely align with the observations made 20 years before in postmortem studies in human brains, leading to the formulation of the hypothesis that dyslexia is a disorder of neuronal migration. As put by Galaburda et al. (2006):

**Table 1:** Functional studies on key dyslexia susceptibility genes

Gene	Study	Method	Species	Comments
*Dyx1c1*	*Evidence in favour of a role in neuronal migration*			
	Wang et al. (2006)	shRNA	Rat	
	Rosen et al. (2007)	shRNA	Rat	Hippocampal malformation
	Threlkeld et al. (2007)	shRNA	Rat	Hippocampal malformation
	Currier et al. (2011)	shRNA	Rat	
	Adler et al. (2013)	shRNA	Rat	
	Szalkowski et al. (2013)	shRNA	Rat	Hippocampal malformation
	*Evidence against a role in neuronal migration*			
	Rendall et al. (2017)	Gene KO	Mouse	
	*Other functions*			
	Threlkeld et al. (2007)	shRNA	Rat	Auditory processing & spatial learning
	Szalkowski et al. (2011)	shRNA	Rat	Working memory
	Szalkowski et al. (2013)	shRNA	Rat	Auditory processing & visual attention
	Chandrasekar, Vesterlund, Hultenby, Tapia‐Paez, and Kere (2013)		Zebrafish	Cilia development/function
	Tarkar et al. (2013)	Gene KO	Mouse	Cilia development/function
	Rendall et al. (2017)	Gene KO	Mouse	Learning & memory
*Dcdc2*	*Evidence in favour of a role in neuronal migration*			
	Meng et al. (2005)	shRNA	Rat	
	Burbridge et al. (2008)	shRNA	Rat	Hippocampal malformation
	Wang et al. (2011)	shRNA	Mouse	(*Dcx* knockdown in *Dcdc2* KO)
	Adler et al. (2013)	shRNA	Rat	
	*Evidence against a role in neuronal migration*			
	Wang et al. (2011)	Gene KO	Mouse	Constitutive and acute KO
	*Other functions*			
	Gabel et al. (2011)	Gene KO	Mouse	Memory & visuo‐spatial perception
	Massinen et al. (2011)		Neuronal cultures	Cilia development/function
	Centanni et al. (2016)	shRNA	Rat	Speech sound discrimination
	Che et al. (2014)	Gene KO	Mouse	Synaptic transmission (slice physiology)
	Truong et al. (2014)	Gene KO	Mouse	Auditory processing & memory
	Grati et al. (2015)		Human cell lines	Cilia development
	Che et al. (2016)	Gene KO	Mouse	Synaptic transmission (slice physiology)
*Kiaa0319*	*Evidence in favour of a role in neuronal migration*			
	Paracchini et al. (2006)	shRNA	Rat	
	Peschansky et al. (2010)	shRNA	Rat	
	Szalkowski et al. (2012)	shRNA	Rat	
	Adler et al. (2013)	shRNA	Rat	
	*Evidence against a role in neuronal migration*			
	Martinez‐Garay et al. (2017)	Gene KO	Mouse	Constitutive and acute KO
	Guidi et al. (2017)	Gene KO	Mouse	Constitutive and acute KO
	*Other functions*			
	Peschansky et al. (2010)	shRNA	Rat	Neuronal branching
	Velayos‐Baeza, Levecque, Kobayashi, Holloway, and Monaco (2010)		Human cell lines	Possible intracellular signalling
	Szalkowski et al. (2012)	shRNA	Rat	Memory & auditory processing
	Szalkowski et al. (2013)	shRNA	Rat	White matter volume
	Centanni et al. (2014)	shRNA	Rat	Neuronal excitability (slice physiology)
	Martinez‐Garay et al. (2017)	Gene KO	Mouse	Prepulse inhibition + anxiety
	Franquinho et al. (2017)	Gene KO	Mouse	Axon growth
			Nuronal cultures	
*Kiaa0319L*	*Evidence in favour of a role in neuronal migration*			
	Platt et al. (2013)	shRNA	Rat	
	*Evidence against a role in neuronal migration*			
	Guidi et al. (2017)	Gene KO	Mouse	Constitutive and acute KO
	*Other functions*			
	Pillay et al. (2016)		Human cell lines	Cell surface receptor
		Gene KO	Mouse	
	Guidi et al. (2017)	Gene KO	Mouse	Auditory processing
*Robo1*	*Evidence in favour of a role in neuronal migration*			
	Andrews et al. (2006)	Gene KO	Mouse	
	Lopez‐Bendito et al. (2007)	Gene KO	Mouse	Interneurons
	Gonda et al. (2013)	Gene KO/shRNA	Mouse	
	Guerrero‐Cazares et al. (2017)		Human neural stem cells	
	*Other functions*			
	Kidd, Lieber, and Young (1998)	Gene KO	Fruitfly	Axon guidance
	Andrews et al. (2006)	Gene KO	Mouse	Axon guidance
	Yeh et al. (2014)	Gene KO/shRNA	Mouse	Cell division

Variant function in any of a number of genes involved in cortical development […] can be responsible for subtle cortical malformations involving neuronal migration and axon growth, which in turn leads to abnormal cortico‐cortical and cortico‐thalamic circuits that affect sensorimotor, perceptual and cognitive processes critical for learning. (Galaburda et al., 2006, p. 1216)

**Figure 3:**
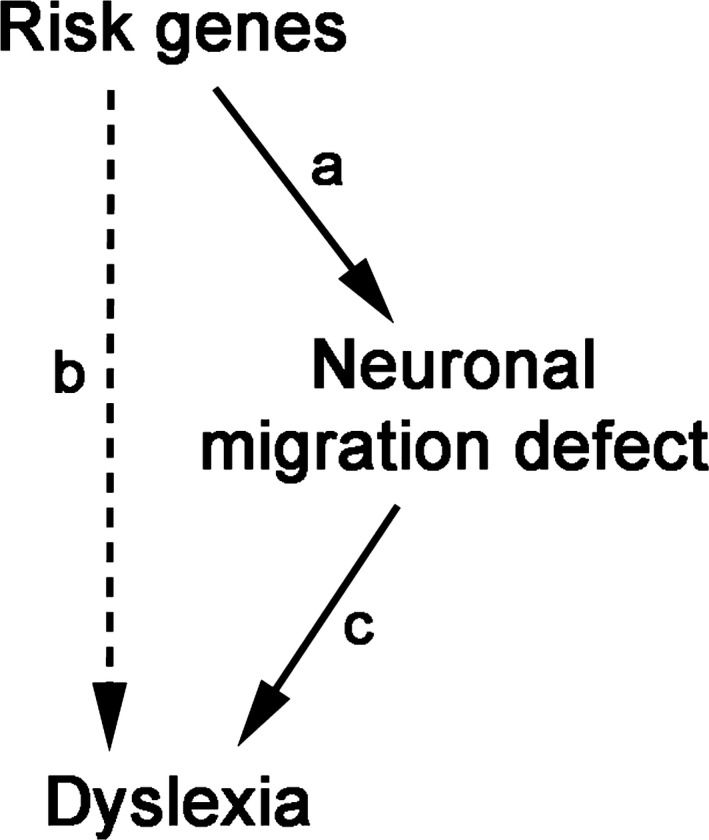
Possible relationship between susceptibility genes and dyslexia. Diagram depicting view where susceptibility genes have a direct causal relationship (solid lines) to dyslexia via defects in neuronal migration (a, c), or one where risk genes lead to dyslexia via a more complex, indirect route (dashed line; b)

The convergence on neuronal migration from the different lines of evidence has established the causal chain illustrated in Figure 3 (path a+c) as the most prominent view on the neurobiological origins of dyslexia. A search on Google Scholar (November 2017) for the combination of terms “neuronal migration” and “dyslexia” yielded around 7,000 returns. The neuronal migration deficit account is currently the most commonly cited hypothesis in the literature on the genetic basis of dyslexia, including citations from our laboratory (Carrion‐Castillo et al., 2013; Fisher & Francks, 2006; Gabel et al., 2010; Giraud & Ramus, 2013; Kere, 2014; Paracchini et al., 2007; Peterson & Pennington, 2015; Poelmans, Buitelaar, Pauls, & Franke, 2011; Raskind, Peter, Richards, Eckert, & Berninger, 2013; Scerri & Schulte‐Korne, 2010; Schumacher, Hoffmann, Schmal, Schulte‐Korne, & Nothen, 2007; Smith, 2007).

Nevertheless, with advances in methods, questions are starting to arise about whether this elegant causal model is correct or other pathways not including neuronal migration are involved (Figure 3, path c). An important part of the focus of our research groups has been primarily on path (a), that is, the link between dyslexia risk genes and disorders of neuronal migration. Doubts about the robustness of this link have arisen at a time when there has also been a reappraisal of path (b), the link between dyslexia and risk genes, which we will also evaluate. Finally, we look more closely at the evidence for path (c), the link between dyslexia and abnormal neuronal migration in humans. We conclude that a strong form of the neuronal migration account where it is the main aetiology of dyslexia is not sustainable. Finally, we suggest an agenda for future research that will allow us to determine whether abnormal neuronal migration plays any role in mediating the link between genetic variants and dyslexia.

### 4.1 The path from dyslexia risk genes to disorders of neuronal migration

#### 4.1.1 Recent advances in functional genetics in mice fail to replicate findings from rat shRNA studies

On the basis of the promising results from rat shRNA studies, several groups, including from our laboratory, started to develop gene‐targeted mice to be used as a tool to gain a more detailed understanding of how these proteins are involved in neuronal migration and in brain function more generally. Knockout (KO) mice were generated for each of the genes mentioned above, carrying mutations to make them unable to produce a normal, functional copy of the protein—the result are animals where the specified protein is never present and, thus, unable to carry out its function inside a cell and in neural circuits. This approach differs from the shRNA method used in rats in that animals completely lack the protein product of a gene from embryogenesis, instead of simply reducing protein levels at the time and place in which the shRNA is introduced. The disruption is bigger and permanent, but requires no intervention during gestation.

**Figure 4:**
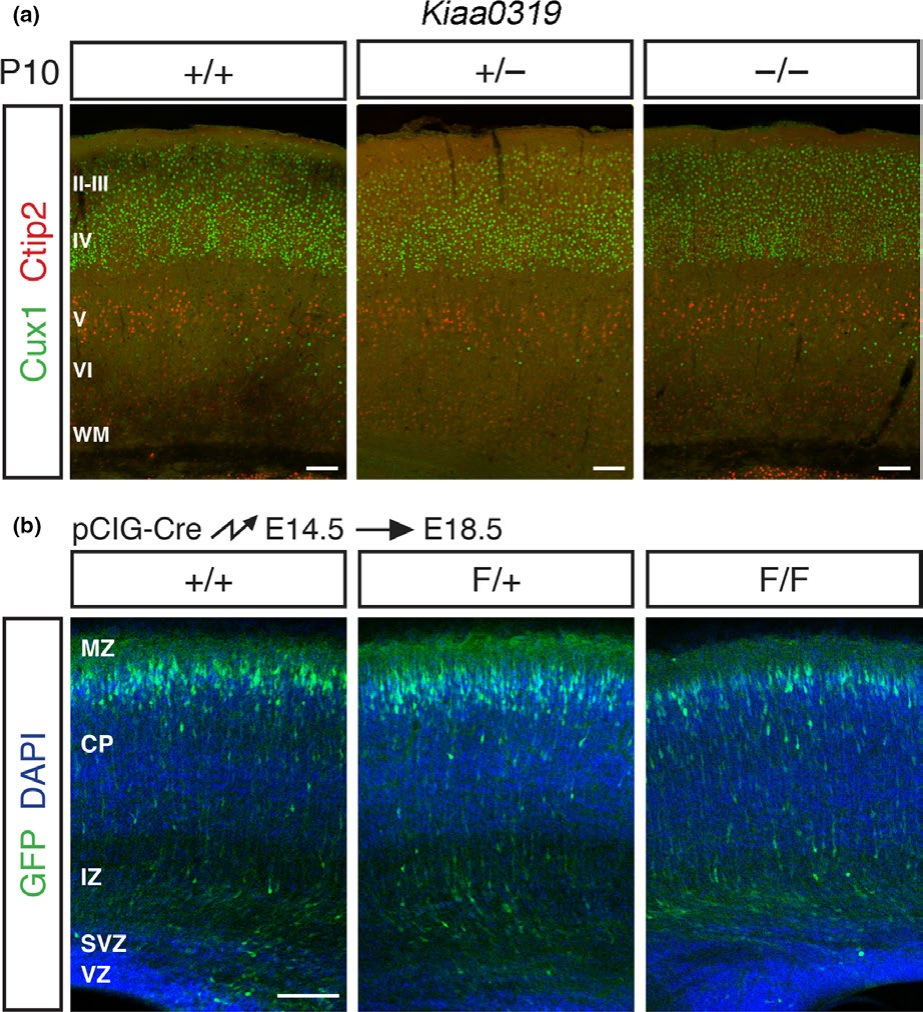
Genetic deletion of KIAA0319 does not affect neuronal migration in mice. (a) Images show sections of the neocortex of mice immunolabelled to identify neurons in the upper layer of the mouse neocortex (Cux1+, II–IV, green) and those in the lower layers (Ctip2+, V–VI, red) for control (+/+), animals lacking one functional copy of *Kiaa0319* (+/−) and *Kiaa0319* KO (−/−) mice at 10 days post‐*partum* (P10). The distribution of the two groups of neurons appears to be the same across the different conditions as they occupy their determined layer, despite the absence of KIAA0319 in the case of mutants, contrary to what would be expected were KIAA0319 to play a role in neuronal migration. (b) Sections of the neocortex of mouse embryos following in utero electroporation with Cre recombinase to disrupt the genetic sequence of *Kiaa0319* and eliminate the production of functional protein. Animals were electroporated at embryonic day E14.5 and analysed 4 days later, using control animals (+/+), mice with one copy of *Kiaa0319* with conditional KO potential (F/+) and mice with both conditional KO copies of *Kiaa0319* (F/F). Cells electroporated are shown in green, with all cells labelled with DAPI in blue. Neurons lacking one (F/+) or both (F/F) copies of *Kiaa0319* (green cells) appear to occupy the cortical plate, near the marginal zone, in the same proportion as that seen in the control sample (+/+), suggesting they were able to migrate as normal. Scale bars = 100 μm. MZ, marginal zone; CP, cortical plate; IZ, intermediate zone; SVZ, sub‐ventricular zone; VZ, ventricular zone. Modified from Martinez‐Garay et al. (2017)

Based on the shRNA knockdown experiments, we would expect that absence of DCDC2 or KIAA0319 proteins, for example, would lead to problems in the migration of neurons during cortical development. However, examination of the brains of each of these KO mice revealed no abnormalities in the organisation of neurons in the neocortex. *Kiaa0319* (Figure 4a; Martinez‐Garay et al., 2017), *Dcdc2* (Wang et al., 2011), *Dyx1c1* (Rendall, Tarkar, Contreras‐Mora, LoTurco, & Fitch, 2017) and *Kiaa0319L* (Guidi et al., 2017) displayed the normal layered structuring in the neocortex and no evidence of layer I ectopias, PVNH or other migration problems, contrary to what would be expected from the shRNA knockdown experiments conducted in rats. These studies are shown in Table 1.

#### 4.1.2 Discrepancies between knockdown and genetic models

Such mismatches between knockdown and knockout methods are well known in the literature on cortical development and other domains (Bai et al., 2003; Corbo et al., 2002; de Nijs et al., 2009; Housden et al., 2017; Pramparo, Youn, Yingling, Hirotsune, & Wynshaw‐Boris, 2010; Young‐Pearse et al., 2007). For dyslexia susceptibility genes, the only gene for which there has been concordance between migration defects in RNAi and KO experiments is *Robo1* (Gonda et al., 2013). In *Dyx1c1*, “constitutive,” complete KOs exhibit major neuroanatomical defects due to severe hydrocephalus resulting from ciliary motility abnormalities (Tarkar et al., 2013) but when *Dyx1c1* was knocked out specifically in the neocortex during its development (using a forebrain‐specific mutant, *Emx1‐Cre*, that targets cortical neurons only), cortical lamination remained unaffected (Rendall et al., 2017). In the case of *Dcdc2* KOs, the layering of the cortex did not display any differences in comparison to wild‐type control mice. It was only when shRNA was used to target the homologous protein DCX that the absence of DCDC2 affected cortical migration: *Dcdc2* KOs displayed a stronger impairment in radial migration following knockdown of DCX than the wild‐type controls (Wang et al., 2011). Doublecortin family members are known to have partially overlapping functions (Deuel et al., 2006; Koizumi, Tanaka, & Gleeson, 2006) and it is possible that the absence of migration defects in *Dcdc2* KOs may be due to compensation by the *Dcx* gene. This could also explain the lack of defects observed at least in *Kiaa0319* or *Kiaa0319L* KOs (Guidi et al., 2017; Martinez‐Garay et al., 2017). But this possibility was ruled out by examining double *Kiaa0319; Kiaa0319L* KO mice (Guidi et al., 2017), where both proteins are fully absent. These mice displayed no evidence of migration abnormalities (see Table 1 for a list of studies).

Different factors can contribute to the discrepancies between knockdown and genetic models. They include compensation in knockout models, distinct dynamics of shRNA versus Cre recombination, potential off‐target effects of shRNA constructs and interspecies differences.

##### Compensation in KO models

Functional overlap between homologous genes is a common source of compensation (Gu et al., 2003; Ohno, 1970), and robustness against null mutations such as those in KO mice is considered a key property of biological systems (Edelman & Gally, 2001; Kitano, 2004). Most genes and proteins do not operate alone and form part of complex gene circuits where degeneracy and redundancy play an important role in buffering against perturbations (whether genetic or not). Via a process of neuronal homeostasis, many cellular and molecular pathways can be activated to ensure a particular process takes place (Ramocki & Zoghbi, 2008). Indeed, a recent study has shown that gene KOs are more likely to activate such compensatory networks by exploiting this plasticity of genetic circuits than knockdown methods, where protein function is disrupted acutely in an otherwise normal system (Rossi et al., 2015). This difference in buffering mechanisms could explain some of the discrepancy observed in phenotypes between the two approaches—in zebrafish, at least, the mismatch in phenotypes seen between genetic mutants and (morpholino) knockdowns has been estimated to amount to around 80% (Kok et al., 2015).

##### Distinct dynamics of shRNA versus Cre recombination

This potential network‐level compensation has been addressed by some of the studies with dyslexia susceptibility candidate genes using mice where a gene can be knocked out in a spatio‐temporal specific manner. In these animals, also called *floxed* mice, the gene in question remains functional throughout development until the point in which DNA is in contact with a protein called *Cre recombinase*. This protein alters the sequence of the target gene and inhibits the production of functional proteins. In the studies mentioned here, DNA constructs expressing Cre recombinase are delivered via in utero electroporation, much in the same way used for the shRNA knockdown experiments performed with rats. The result at the genetic level is the same as in constitutive KOs, except for the different time points of disruption and the proportion of cells affected—constitutive KOs target all cells, while the conditional method only disrupts gene function in the cells transfected with the Cre construct. Apart from the species difference, the two methods differ only with respect to the molecular stage where gene function is disrupted—targeting mRNAs in the case of shRNA, or DNA in floxed mice. This approach recapitulates in mice the same developmental conditions of the shRNA knockdown experiments that originally linked the genes in question to neuronal migration, and it circumvents potential network‐level compensation that may occur in constitutive KO mice. However, it is important to consider that the dynamics of protein knockdown will differ between the two systems, with shRNA providing a faster decrease than Cre‐mediated recombination.

Experiments have been conducted with this method to interrogate the function of *Dcdc2*,* Kiaa0319* and the joint effect of *Kiaa0319* and *Kiaa0319L*: the acute disruption did not lead to observable problems in migration in any of the three cases (Figure 4b; Guidi et al., 2017; Martinez‐Garay et al., 2017; Wang et al., 2011). These results are in stark contrast to the findings obtained with shRNA for each of these genes (Meng et al., 2005; Paracchini et al., 2006; Platt et al., 2013). Although the differential dynamics between shRNA and Cre recombinase protein knockdown could partly explain these discrepancies, the magnitude of the difference between the results obtained by the two approaches for three separate genes makes this explanation highly unlikely. In the case of *Dyx1c1*, mice with conditional knockout potential are available (Rendall et al., 2017) but there have been no reports showing the effects when using this approach.

##### Off‐target effects in shRNA knockdown

Although it is possible that rat‐mouse species differences may be implicated (see below), there are strong reasons to believe that discrepancies in results are likely to derive from off‐target effects triggered by the use of shRNA. RNA interference approaches have been a powerful tool for functional genetics and are widely used, but specificity has been a constant source of concern and a point of investigation over the years (see e.g., Grimm et al., 2006; Housden et al., 2017; Jackson & Linsley, 2010). More importantly, it has been recently shown that shRNA‐mediated knockdown of DCX leads to deficits in migration that are indistinguishable when performed in wild‐type animals and in *Dcx* KO mice, where no *Dcx* mRNA is present (leaving no target for the shRNA; Baek et al., 2014). Work on the schizophrenia susceptibility candidate gene *Disc1* has revealed similar results, as cells electroporated with *Disc1*‐shRNA vectors fail to migrate even in *Disc1* KO brains (Kvajo, McKellar, & Gogos, 2012; Tsuboi et al., 2015). Although dosage can play a significant role in the triggering of off‐target effects (Caffrey et al., 2011), several other reports have been published with parallel effects following the use of shRNA (Alvarez, Ridenour, & Sabatini, 2006; McBride et al., 2008). But what then causes the migration abnormalities observed in these studies? In the example of *Dcx* (Baek et al., 2014), it was shown that shRNAs can lead to a disruption in the levels of microRNAs which, in turn, can cause problems with cell migration (see also Grimm et al., 2006). While genome‐editing approaches also have drawbacks, such specificity problems are a major issue with RNA‐based methods (Housden et al., 2017).

##### Interspecies differences

The study by Baek et al. (2014) also serves as comparison for the potential differences across species. In humans, null‐mutations in the *DCX* gene can cause profound defects in cortical migration (Gleeson et al., 1998), and acute knockdown with shRNA in rats leads to parallel abnormalities (Bai et al., 2003), but mice carrying similar mutations do not display similar problems (Corbo et al., 2002; Pramparo et al., 2010). The same has been found for the *LIS1* gene (Reiner, 2013). In the studies with dyslexia‐susceptibility genes in rodents mentioned above, shRNA knockdown has been performed exclusively in rats and genetic KO (constitutive or conditional) only in mice. Thus, it is possible that the discrepancies observed may result in part from differences across these two rodent species. So, could mouse‐rat differences be responsible for the discrepant findings? This seems unlikely because when shRNA was used in mice for other genes, such as *Dcx* and *Disc1*, knockdown led to neuronal migration deficits that mirrored those obtained in rats (Baek et al., 2014; Bai et al., 2003; Kvajo et al., 2012; Ramos, Bai, & LoTurco, 2006; Tsuboi et al., 2015). Based on these results and the issues with specificity of shRNA mentioned above, and because of the lack of rat KO models for dyslexia susceptibility genes, the most parsimonious explanation would be that off‐target effects are implicated in the results obtained with both mice and rats. However, a more important question that derives from this analysis is the degree of interspecies differences between humans and rodents. How much can we translate a lack of migration defects obtained in mice to the human brain? The DCX and Lis1 examples highlight that mutations in the same genes in mice and humans do not necessarily lead to the same phenotype despite conserved molecular migration mechanisms. In fact, it has been shown that the effect of null mutations in mice can lead to significantly different phenotypes to what is seen in humans (Liao & Zhang, 2008). Thus, size (the distance neurons are required to migrate in the prenatal human brain is considerably longer than in the mouse brain) and complexity of the human brain are probably the main factors underlying these differences.

#### 4.1.3 Functional genetics of dyslexia and neuronal migration

What does this mean for our understanding of the functional genetics and neurobiology of dyslexia? Experimental methods are almost always imperfect or offer only indirect ways of interrogating the desired variables—because of this, specific findings must be demonstrated using more than one method so as to reduce the probability that observations are spurious or result from the experimental manipulation per se (Popper, 1934). The association between dyslexia susceptibility genes and neuronal migration was shown using one method, but has not been confirmed with an alternative technique. The body of evidence outlined above raises questions about whether the original results are due to real modulation of gene function or methodological artefacts. It is possible that future evidence shows this relationship to hold but, on the face of the current evidence base, the putative link between these genes and neuronal migration lacks evidential support and can no longer be mentioned without a statement of the known inconsistencies.

### 4.2 The association between common genetic variants and dyslexia

The evidence from functional genetics is based on studies conducted on 4 to 5 candidate genes: *KIAA0319*,* DCDC2*,* DYX1C1*,* KIAA0319L* and, to some extent, *ROBO1*. Although these genes correspond to the strongest candidates, they only explain a small fraction of the genetic component underlying dyslexia and are likely to be a small subset of genes implicated in susceptibility to a complex, heterogeneous disorder like dyslexia.

The identification of these genes as susceptibility candidates was based primarily on the use of fine‐mapping and positional cloning studies that were prevalent around the early 2000s. However, these methods precede the use of genome‐wide approaches, be it GWAS or next generation sequencing (NGS), which have revolutionised molecular genetics and our understanding of the genetic architecture of complex disorders. While similar work on disorders of language is still in its early stages (Graham & Fisher, 2015; Newbury et al., 2014; Paracchini et al., 2016), other neurodevelopmental disorders such as autism and schizophrenia have been shown to involve over a hundred risk variants which can vary in frequency (common vs. rare) and phenotypic penetrance (small vs. big effects; see e.g., Mitchell, 2012; Bourgeron, 2015). The number of genes implicated varies in the degree of confidence of association but is in the order of hundreds—for example, the database AutismKB lists over 3,000 candidate genes for autism, of which 150 are considered as high‐confidence candidates (Xu et al., 2012), and the latest update from April 2018 of the database SFARI Gene lists over 1000 candidate genes for autism, of which only 25 are considered as high‐confidence candidates (Abrahams et al., 2013). It is likely that a similar picture will emerge for the genetic architecture of dyslexia as research continues (Graham & Fisher, 2015). As we advance in our understanding of the genetics of dyslexia, it may be that they become only marginal, historical candidates in the long run, much in the same way that has happened with other disorders, such as *DISC1* in schizophrenia (Mitchell, 2012). In particular, it has been shown that, in the context of GWASs, candidate genes for schizophrenia do not show stronger signals than non‐candidates (Johnson et al., 2017). Nonetheless, important challenges remain in neurogenomics more generally, not only in the study of language, as highlighted by Mitchell (2018).

It has to be noted that a major limitation of genome‐wide investigations for dyslexia is the relatively small sample size analysed so far which is in the range of a few thousands and is not sufficient to give adequate power to detect the expected small size effects (Park et al., 2010). Furthermore, we cannot refer to them as GWASs for dyslexia because they often test for genetic associations with reading abilities in the normal range of variation using general population samples (see Newbury et al., 2014; Paracchini, 2011). Although genome‐wide approaches to language disorders are not without their challenges, a growing body of work is starting to uncover new genes putatively implicated in dyslexia and these are associated with a range of different neurodevelopmental and neuronal functions such as regulation and function of ion channels, glucose transport, synaptic plasticity, and so on (Graham & Fisher, 2015; Newbury et al., 2014; Paracchini et al., 2016).

Recent functional studies are shedding new light onto the function of the classical susceptibility genes. *DCDC2*,* DYX1C1* and *KIAA0319* are highly expressed in ciliated tissues (Ivliev, t Hoen, van Roon‐Mom, Peters, & Sergeeva, 2012). Knock‐down studies of *Dyx1c1* in zebrafish and mouse confirmed a role in ciliogenesis (Tarkar et al., 2013) while *Dcdc2* was found to regulate the length and function of cilia (Massinen et al., 2011). When disrupted, *DYX1C1* and *DCDC2* cause ciliopathies (Schueler et al., 2015; Tarkar et al., 2013). The already mentioned *CEP63*, identified by exome sequencing in a large family with dyslexia, is required for cilia formation (Einarsdottir et al., 2015). These findings have led to the suggestion of a role of primary cilia in underlying dyslexia susceptibility (Brandler & Paracchini, 2014; Kere, 2014; Paracchini et al., 2016). While it remains possible that cilia could mediate neuronal migration, it is interesting to note that, for the patients with ciliopathies carrying *DYX1C1* and *DCDC2* mutations, symptoms of dyslexia or other cognitive problems have not been reported. In addition, DCDC2 has been reported to be involved in synaptic transmission (Che, Girgenti, & LoTurco, 2014; Che, Truong, Fitch, & LoTurco, 2016), KIAA0319 in axon growth (Franquinho et al., 2017) and KIAA0319L as a cell surface adenovirus receptor (Pillay et al., 2016). In addition, the zebrafish homolog of *KIAA0319* has been recently found to be expressed in several structures other than the brain (otic vesicles, eyes and notochord), thus suggesting other functions (Gostic et al., 2018). It is possible that some of these processes may influence neuronal migration but convincing evidence is still lacking. A list of studies describing cellular functions of the main dyslexia candidate genes in neuronal migration and beyond is shown in Table 1.

Another open question is how it is possible for a general process such as neuronal migration to specifically affect dyslexia. From a genetic point of view, it has to be considered that the variants associated with dyslexia predominantly fall within regulatory regions (thus, affecting levels of expression rather than the function of a gene), in line with what is known for most other complex, multifactorial traits. As such, it is unlikely that risk variants in genes such as *KIAA0319* or *DCDC2* are sufficient to lead to defects in neuronal migration or other neurodevelopmental pathways contributing to dyslexia—particularly given some of these risk variants are also commonly found in non‐dyslexia populations—and thus must co‐occur with other factors. This common misconception is one of the problems underlying many brain imaging or behavioural studies for neurodevelopmental traits in general. With specific reference to dyslexia, it has been assumed that common genetic variants such as those seen in *DCDC2* have a large effect size, justifying analyses using very small samples, eventually leading to identification of false positives (for a recent study highlighting these issues see Scerri et al., 2017).

### 4.3 The association between dyslexia and neuronal migration abnormalities in humans

The hypothesis that dyslexia is a disorder of neuronal migration was originally based on postmortem neuropathological examinations of dyslexic brains. We should start by noting that the analyses of cortical structure performed in the original reports by Galaburda and colleagues were based on high standards and thorough examination of each of the brains (which were sectioned at 35 μm and every 20th examined). This meticulous examination of the samples available with serial analyses of sections was ground‐breaking at the time, and indeed went beyond current practice in modern human neuropathology. Most contemporary histopathological work involves investigation of only a few selected areas of the brain (see e.g., McKavanagh, Buckley, and Chance (2015), where four BA regions are studied), not across the rostro‐caudal length as in the dyslexia studies. Nevertheless, there are important issues with the postmortem analyses in human samples, most of which have been raised elsewhere but received little attention in the literature (Altarelli et al., 2014; Beaton, 1997).

First, there are doubts over how representative of dyslexia the samples examined are. It should be stressed that postmortem brain material from dyslexic individuals is exceedingly rare, researchers typically have little control over clinical evaluation of patients whose brain come for analysis, and it is often inevitable that there will be limited information about how the diagnosis was made. The original case of Drake (1968) was an exception, as the child died soon after detailed psychological and cognitive assessments had been conducted. These confirmed he had normal IQ and reading difficulties, but also indicated a host of other issues: serious attentional, emotional and behavioural problems, as well as recurrent headaches and what sound like possible absence seizures: “lapses of attention with staring into space, and ‘dizzy spells’ with ‘blackouts’” (p. 487). As noted by Altarelli et al. (2014), the female cases examined in another study (Humphreys et al., 1990) display co‐morbidity with other neurological conditions which may confound the observations. The authors note that, of the three patients studied in the report, the first patient suffered from severe depression and attention deficits, while patient 3 had delayed language acquisition and was suspected for ADHD. A further problem lies with patient 2 never having received formal psychological assessment, leaving the extent of the reading disability unknown and the possibility of other conditions open. Diagnostic problems have also been noted (Beaton, 1997) for the male patients reported in Galaburda et al. (1985). With respect to the first case reported, the authors point out in Galaburda and Kemper (1979) (p. 94) that the patient developed nocturnal seizures at the age of 16 years and he had delayed speech development. Case 2 in Galaburda et al. (1985) also presents a profile that goes beyond the typical assessment for dyslexia as the patient had notable language difficulties and received special education.

If we take these considerations into account, only three of the nine samples investigated could be considered free from other conditions. This does not necessarily invalidate a dyslexia diagnosis: co‐morbidity is expected given how commonly dyslexia co‐occurs with other disorders, especially with delayed language and speech development or DLD (see e.g., Bishop, 2015; Newbury et al., 2011). However, where there are comorbidities, it is difficult to know which aspect of the clinical presentation is related to neuropathological abnormalities. Epilepsy is a particularly challenging confound, given that neuronal migration abnormalities are often a focus for seizures (Lee et al., 2001). This does not mean these samples must be discarded; rather, co‐morbidity must be carefully controlled for in such studies.

Viewed through a contemporary lens, the main limitation of the early studies was that the analyses were not blinded (Lazic, 2016): initially there was no control group and non‐dyslexic samples were examined and reported separately (Kaufmann & Galaburda, 1989). The ideal would be to have a control group of brains, matched for age and gender (including non‐dyslexic cases affected by the same comorbid conditions as dyslexics), with the analysis done without awareness of which group the brain came from and following modern standards of postmortem human neuropathology (e.g., Adorjan et al., 2017). Blind experimental design is particularly important when examining microscopic details such as dyslamination and ectopias across a large number of sections.

What about the other source of evidence—the studies that turn the question on its head and look at reading abilities in patients with PVNH? (Chang et al., 2005, 2007). On further inspection, the evidence presented from these studies is suggestive but not compelling, with experimental design again less than optimal. In the study by Chang et al. (2005), a consecutive series of 10 patients with PVNH and epilepsy were evaluated. Nine of the 10 had normal range IQ, and two had been formally diagnosed with dyslexia or a language‐based disability in the past. On the Wide Range Achievement tests of reading and spelling, the mean scores were average or above‐average. Many of the patients did, however, do poorly on the Nelson‐Denny reading test and, on this basis, the authors concluded they were dyslexic. But this test, which stresses speed, was designed for college students, not for the general population. The fact that most participants were older than college students, and all were on anti‐epileptic medication, makes the claim of dyslexia in these people far from convincing. The 2007 study had a better design: 10 patients with PVNH were compared with 10 dyslexics and 10 adults without dyslexia. Nevertheless, the groups were not well matched: the normal readers were recruited through local universities and had a mean age of 25.5 year, 10 years younger than the other two groups. It would have been preferable to use another patient group, or relatives of PVNH patients, to achieve a more closely matched comparison group against which to evaluate the patients. The PVNH patients (who included five cases seen in the 2005 study) once again did poorly on speeded reading tests (as did the dyslexics), and were unimpaired on untimed reading (which the dyslexics also were unimpaired on, rather surprisingly). However, on a phonological awareness test, only the dyslexics were impaired. In this regard, the PVNH patients did not have a classic dyslexic profile. An additional correlational analysis of white matter fractional anisotropy and a rapid naming measure in six PVNH cases is not statistically significant when appropriate corrections are applied for multiple testing. One thing that is clear from these studies, and consistent with others with this patient group (Dubeau et al., 1995), is that outcomes are very diverse. The key question is not so much what the average reading ability is, but whether there is an increased risk of dyslexia, and if so, whether it is predictable from the PVNH characteristics. This is not possible to establish from the published cases to date. A further study of 10 children with PVNH (Felker, Walker, Sokol, Edwards‐Brown, & Chang, 2011) suffered from similar limitations: although a control group was used, they were from word‐of‐mouth referrals and had a mean IQ 20 points higher than the PVNH cases, four of whom were on anti‐epileptic medication. Six of the PVNH cases were reported to have a history of reading problems, and three of these received special education, but the presentation of the data as group means makes it difficult to establish their specific cognitive profile.

Perhaps the most important piece of evidence from human studies is the absence of associations reported between neuronal migration abnormalities and dyslexia from brain imaging studies. One is reminded of the incident in a Sherlock Holmes story where a mystery was solved by observing that a dog did not bark in the night during the theft of a racehorse (Conan Doyle, 1893). Since the studies by Chang et al. (2005, 2007), there appears to be only one further case linking PVNH and reading impairment (Reinstein, Chang, Robertson, Rimoin, & Katzir, 2012) despite hundreds of MRI images of dyslexic brains taken for other studies—for example, a meta‐analysis by Jednoróg et al. (2015) included 236 cases. This negative evidence is not conclusive, since PVNH may be missed when not actively searched for. It is also possible that smaller abnormalities in cortical organisation resulting from neuronal migration deficits such as ectopias and dyslamination may be present in these samples but are simply too small to be detected with the field strength used in these in vivo neuroimaging studies. It has been long argued that cortical neuronal migration defects can be subtle and underestimated because of the differences in timing in different cortical areas (Rakic, 1988). It is the case that a number of studies have reported that polymicrogyria, another type of cortical migration abnormality, appears to be enriched in individuals with more general impairment in language, not specific to reading (Guerreiro et al., 2002; Hage, Joaquim, Carvalho, Padovani, & Guerreiro, 2004; Leventer et al., 2010; Oliveira et al., 2008; Webster et al., 2008), though this is not a common finding in children with DLD (Morgan, 2013).

## 5 CONCLUSION AND FUTURE DIRECTIONS

The neuronal migration hypothesis of dyslexia is based on two key lines of evidence: functional genetics on a handful of susceptibility candidate genes in rodents, and postmortem histopathology in human dyslexia cases. In this review, we outlined a number of issues surrounding both of these points which, altogether, question the strength of the evidence in favour of the neuronal migration view. We make the case that this position is untenable in the face of our current knowledge of the function of candidate genes studied so far, the genetic architecture of dyslexia and human neuropathology, unless the original findings are replicated using modern standards. Reproducibility is one of the key tenets of scientific research and there has been growing concern over its status in biomedical research in recent years (Begley & Ioannidis, 2015; Bustin, 2014; Munafò et al., 2017). When the first functional genetics results from in utero electroporations in rat embryos emerged, the convergence between those experiments and its parallels to human studies were remarkable, generating a great deal of excitement in the language sciences community.

It is now time to become equally interested in engaging with the shortcomings of our own work and build on it so as to keep advancing our knowledge of the neurobiology of language and reading in the normal and diseased brain. At the cellular level, recent work has started to uncover new players and processes involved in dyslexia susceptibility, from axon growth (Franquinho et al., 2017) and modulation of synaptic transmission (Che et al., 2014, 2016), to the structure and function of primary cilia (Brandler & Paracchini, 2014; Kere, 2014; Paracchini et al., 2016). The identification of novel candidate genes shall also elicit new evidence and contribute to efforts to uncover other biological pathways. One important part of this debate is whether work conducted so far has been based on the best possible models—i.e., are rodents the best organisms to understand abnormalities of language and reading or should we be looking at alternatives? There are several examples of work in the language sciences using other species such as bats (Vernes, 2017), songbirds (Bloomfield, Gentner, & Margoliash, 2011; Fisher & Scharff, 2009; Prather, Okanoya, & Bolhuis, 2017), non‐human primates (Hage & Nieder, 2016; Takahashi et al., 2015), and so on (Fitch, 2017; Kiggins, Comins, & Gentner, 2012). Further work in human cell lines will also be important to understand the molecular function of candidate genes. Genome technology might identify rare variants contributing to dyslexia or within candidate genes for dyslexia in individuals with different conditions which might shed light on the function of these genes.

We do not question that disrupted neuronal migration can have important consequences for cognitive development in humans. The question is how far this specific aetiology is implicated in causing dyslexia, and how specific an aetiology it is. To address the first question, we need studies that use the latest technological and statistical advances in neuroimaging, such as variations and improvements (e.g., 7T MRI) to identify subtle cortical malformations in large and well‐documented series of individuals with dyslexia (Hong et al., 2014; Pardoe & Kuzniecky, 2014; Wang et al., 2015). This will help establish the prevalence of disorders of neuronal migration as a causal factor. In addition, to address the second question, there is a need for studies in the reverse direction, to look at the outcomes of individuals with features such as ectopias. These need to give careful consideration to design features such as selection of appropriate control groups and blinding of experimenters. We already know that the same genetic mutation can have remarkably variable impact on neurodevelopment (Wilson et al., 2017). We anticipate that the same may be true of abnormalities of neuronal migration.

## CONFLICT OF INTEREST

Authors declare no conflict of interest.

## AUTHOR CONTRIBUTIONS

LGG, AVB, SP, DVMB and ZM drafted the manuscript. IMG and APM revised the original draft and provided additional input. All authors critically revised the manuscript and approved its final version.
