# Supplementary material for: The neuronal migration hypothesis of dyslexia: A critical evaluation 30 years on
Source: Eur J Neurosci. 2018 Oct 6;48(10):3212–33. doi: 10.1111/ejn.14149 (PMC6282621; doi:10.1111/ejn.14149)
Supplement: Supplementary file 1 [file EJN-48-3212-s001.docx]

The Neuronal Migration Hypothesis of Dyslexia: A Critical Evaluation Thirty Years On

Molnar, Zoltan; Guidi, Luiz; Velayos-Baeza, Antonio; Martinez-Garay, Isabel; Monaco, Anthony; Paracchini, Silvia; Bishop, Dorothy

_____________________________________________________________________________________

| Review timeline: | Submission date: 15/05/18  Editorial Decision: Minor Revision  Revision Received: 06/08/18  Accepted: 13/08/18 |  |
| --- | --- | --- |
|  |  |  |

Editor: Sophie Molholm

Reviewer 1: Nenad Sestan

Reviewer 2: John Foxe

_____________________________________________________________________________________

1st Editorial Decision	29/05/18

_____________________________________________________________________________________

The Neuronal Migration Hypothesis of Dyslexia: A Critical Evaluation Thirty Years On

Dear Prof. Molnar,

Your manuscript was reviewed by two  reviewers as well as by the Section Editor,  Dr. Sophie Molholm, and ourselves. Both reviewers are very positive about your manuscript and have only minor comments for strengthening the paper.  We apologize for the delay in getting the paper reviewed.  We were finally able to secure a second qualified reviewer with the time to perform the review.  We ensure you that turn around will be quick once you have addressed the reviewer comments.  We look forward to receiving your revised manuscript.

When revising the manuscript, please embolden or underline major changes to the text so they are easily identifiable and DO NOT leave 'track change' formatting marks in your paper.  Please ensure that you provide a text and a figure file for the Graphical Abstract (as detailed in the instructions below).  When carrying out your revisions please refer to the checklist below and visit the EJN author guidelines at www.ejneuroscience.org

When finalized, please upload your complete revised manuscript onto the website, as a Word file (.doc, or .docx).  Please also ensure that a complete set of tables and figures is included as separate files, even if these have not changed from the originals.  At this stage it is necessary to provide high resolution figures.  Please see important instructions below.

Please go into https://mc.manuscriptcentral.com/ejn - Author Centre - manuscripts with decisions where you will find a 'create a revision' link under 'actions'. We ask that you please indicate the way in which you have responded to the points raised by the Editors and Reviewers in a letter. Please upload this response letter as a separate Word (.doc or PDF) file using the file designation "Authors' Response to Reviewers" when uploading your manuscript files.  Please DO NOT submit your revised manuscript as a new one.  Also, please note that only the  Author who submitted the original version of the manuscript should submit a revised version.

If you are able to respond fully to the points raised, we would be pleased to receive a revision of your paper within 30 days.

Thank you for submitting your work to EJN.

Kind regards,

John Foxe & Paul Bolam
co-Editors in Chief, EJN


Reviews:

Reviewer: 1

Comments to the Author
The authors. evaluated recent experimental evidence not supporting the link between dyslexia and neuronal migration. By examining gene function studies of rodent models as well as focusing on human histopathological and imaging studies, the authors summarize that neuronal migration hypothesis of dyslexia is not solid, highlighting the importance of new future technology and animal models.

This is an outstanding review.  Despite my strong enthusiasm, I do feel the manuscript could benefit from some minor edits, which I leave at the discretion of the authors.

Minor concerns:
1. I think the authors should elaborate more on interspecies differences. They should point out that the distance that neurons has to migrate can be a few hundred time longer in the prenatal human brain compared to rodent brain. Also, the author should consider citing a study by Liao and Zhang PNAS 2008 (PMID: 18458337), which has shown that null mutations in human and mouse orthologs frequently result in different phenotypes.

2. While some description of shRNA knockdown technology is necessary to make the review accessible to a wide audience, the authors could be more concise in discussing the artefacts and technical issues associated with shRNA technology and differences between that and knock-out models.


3. As the authors have written this review, they seem to suggest that the most important line of evidence for evaluating the association between dyslexia and neuronal migration abnormalities comes from human brain imaging. However, the authors only give limited consideration and explanation of the results from MRI, as well as the pitfalls of this methodology.

4. Though GWAS for dyslexia is hard because of its relatively small sample size and heterogeneous cohort, it’s still better for the authors to compare the available genome-wide investigation results with classic functional genetics studies, which may guide future researchers about new candidate genes and alternative mechanisms.

5. Page 6 line 6, how many patients were included in Galaburda et al.’s study? As to compare with the disease group, detailed number of cohort, as well as percentages of abnormalities in language-related areas are needed.

6. In Fig.3, the indicated dashed line according to the legend is a solid line.

7. Page 11 line 50, referring to other neurodevelopmental disorders such as ASD and schizophrenia, there are much more recent databases and publications instead of the Xu et al., 2012 paper.


Reviewer: 2

Comments to the Author
I thoroughly enjoyed reading this extremely well-constructed, clearly articulated, and ultimately sobering review article. It could be published “as is” in my view, so the following comments are purely meant as potential things to be considered and are entirely of the minor variety.
- Page 4, Line 27: Is the VWFA (poorly named – see e.g. Vogel, Petersen, and Schlaggar, 2014) really unilateral?
- One observation from our own work is that when considering connectivity models of reading, one might also need to consider what the task of learning to read involves – that is, the association of already learned phonological information with orthographic inputs that children only receive exposure to after language has already emerged. That is, the kids need to learn a large set of visual-auditory cross-sensory associations and this must necessarily involve communication between visual and auditory regions/representations (see Hahn, Foxe and Molholm, 2014). I realize this is not central to the arguments being made in this review and mention it here only as an aside.  However, this observation goes to something I note in the paper/field as a whole, and that is that the actual human deficit is not often considered in detail when animal model work is being conducted. This also goes to the early days of this work when the neuronal migration thesis was first emerging. It is a puzzle why migratory issues were assumed to only affect reading but not the basic language functions of these areas.
- On page 11 (lines 28 and 29), I was struck by the concluding statement, which seems altogether too accommodating of the prior work. The authors have really made a quite convincing argument by this stage that such a link has little to no convincing evidence and may wish to strengthen this statement.
- Bottom of page 11: I would point the authors to Kevin Mitchell’s recent EJN editorial on the candidate gene approach, which seems highly germane to this section of the paper -- https://onlinelibrary.wiley.com/doi/abs/10.1111/ejn.13801

Typographic:
Page 5, line 35: “neurons ectopias” needs to be corrected
Page 6, line 1: Pluralize “dysgenesis” – “dysgeneses”
Page 6, Line 6: “method” should be “methods”
Page 7, line 41: I would suggest replacing “mirror in” with “closely align with”
Page 8, line 3: Give date of Google Scholar search
Page 12, line 20: “lights” should be “light”
Page 12, line 26: Remove “study” where it says “exome sequencing study”
Page 14, line 5: Shouldn’t “Viewed from a … lens” be “Viewed through a … lens”
Page 15, lines 26-27: “on the face” should probably read “in the face”
Page 15, line 43 “efforts of uncovering” should probably read “efforts to uncover”

------------------------

____________________________________________________________________________________

Authors’ Response	06/08/2018

_____________________________________________________________________________________

The Neuronal Migration Hypothesis of Dyslexia: A Critical Evaluation Thirty Years On

Dear Professors Foxe and Bolam,

Thank you very much for sending us the reviews for our manuscript. We are very grateful to Section Editor, Dr. Sophie Molholm and to you both of you for constructively criticizing our review that helped us to further strengthen our paper. Thank you for ensuring us that turn around will be quick after we have addressed the reviewer comments.

We uploaded our revised manuscript onto the website, as a Word file (.doc, or .docx). We included the tables as separate files, and we provide high resolution figures.

We embolden the major changes to the text so you can easily identify the chances.

We also provided a text and a figure file for the Graphical Abstract.

We listed all the changes and described how we have responded to the points raised by the Editors and Reviewers in a letter. This is uploaded as a separate response letter (.doc) file using the file designation "Authors' Response to Reviewers".

We are looking forward to hearing from you in due course.

Kind regards,


Zoltán Molnár MD DPhil

Antonio Velayos-Baeza PhD

We have addressed all comments from the reviewers as detailed below. This involved the inclusion of five new references and the removal of one from the Reference list; details are provided in the responses to the relevant comments. The parts of the text that have been modified are shown in **bold**, except for several minor typographic corrections and changes in references and citations. Furthermore, while revising the manuscript, we included a new citation in current p. 27 (line 6 in the last paragraph) to improve the text; this extra reference (Reinstein et al., 2012) has been added to the Reference list.

We have also adapted the format of our files to the required specifications of Eur J Neurosci; this included the use of double spacing, removal of line numbers, minor modifications in reference listing and inclusion of some information in the Title page. This means that the references to page and line numbers made by the reviewers in several of their comments have now changed, but specific indications of the location of the modifications made in the revised version are provided in the responses to each comment.

**Reviewer: 1**

Comments to the Author

The authors. evaluated recent experimental evidence not supporting the link between dyslexia and neuronal migration. By examining gene function studies of rodent models as well as focusing on human histopathological and imaging studies, the authors summarize that neuronal migration hypothesis of dyslexia is not solid, highlighting the importance of new future technology and animal models.

This is an outstanding review. Despite my strong enthusiasm, I do feel the manuscript could benefit from some minor edits, which I leave at the discretion of the authors.

Minor concerns:

1. I think the authors should elaborate more on interspecies differences. They should point out that the distance that neurons has to migrate can be a few hundred time longer in the prenatal human brain compared to rodent brain. Also, the author should consider citing a study by Liao and Zhang PNAS 2008 (PMID: 18458337), which has shown that null mutations in human and mouse orthologs frequently result in different phenotypes.

Response: We thank the reviewer for the comment on the “migration distance” and for pointing out this important reference, and have modified the end of section 4.1.2.4, now at the end of p. 19, to address both points as follows: “In fact, it has been shown that the effect of null mutations in mice can lead to significantly different phenotypes to what is seen in humans (Liao & Zhang, 2008). Thus, size (the distance neurons are required to migrate in the prenatal human brain is considerably longer than in the mouse brain.” The cited reference has been added to the Reference list.

2. While some description of shRNA knockdown technology is necessary to make the review accessible to a wide audience, the authors could be more concise in discussing the artefacts and technical issues associated with shRNA technology and differences between that and knock-out models.

Response: We recognise that the explanation is comprehensive, but feel that this is one of the strengths of the manuscript and that a clear, accessible explanation for this lies at the heart of making the argument convincing to the widest possible audience. It is only by not obscuring these details that we can demonstrate the inconsistency of the evidence supporting the neuronal migration hypothesis. Thus, we would like to leave this part of the manuscript (end of 2^nd^ paragraph of section 4, now in p. 11) without any further modification.

3. As the authors have written this review, they seem to suggest that the most important line of evidence for evaluating the association between dyslexia and neuronal migration abnormalities comes from human brain imaging. However, the authors only give limited consideration and explanation of the results from MRI, as well as the pitfalls of this methodology.

Response: We discuss evidence of neuronal migration abnormalities from MRI studies at the end of section 4.3 and in section 5, now on pp. 27-30, noting that this is a particularly important source given the *absence* of relevant evidence. There are numerous studies examining structural abnormalities in dyslexia samples using MRI but these cover a range of other types of deficits such as asymmetry of the planum temporale or gray matter volume, without reference to problems related to neuronal migration. We point out that this could be due to limitations of the methodology, given that dyslamination and ectopias may be too small to be detected using current MRI methods, and highlight (p. 29) the fact that newer methods (e.g. 7T MRI) may allow this to be overcome.

4. Though GWAS for dyslexia is hard because of its relatively small sample size and heterogeneous cohort, it’s still better for the authors to compare the available genome-wide investigation results with classic functional genetics studies, which may guide future researchers about new candidate genes and alternative mechanisms.

Response: We entirely agree with the reviewer that we should make the most of all available evidence on susceptibility genes for dyslexia, be it from functional studies or GWAS. In fact, we highlight a number of genes which have been reported for possible association to dyslexia (end of section 2, pp.7-8) as well as how the ‘classic’ candidate genes relate to them (section 4.2, pp. 20-21). However, to the best of our knowledge, no candidate gene suggested from GWAS so far has been shown to be involved in neuronal migration and, thus, these candidates do not offer direct evidence in relation to the neuronal migration hypothesis – in fact, this offers further indication of the limited evidence base for this view, a point we make in p. 22. We think including here any functional information about the genes detected by GWAS would add unnecessary complexity to this review and distract from the focus on neuronal migration.

5. Page 6 line 6, how many patients were included in Galaburda et al.’s study? As to compare with the disease group, detailed number of cohort, as well as percentages of abnormalities in language-related areas are needed.

Response: The number of samples was indicated in brackets (3 out of 10) but we recognise the information was not presented in the most salient way. We have edited this sentence in the 2^nd^ paragraph of section 3 (now in p. 9) to this effect as follows: “Using their rigorous serial section method, they found that abnormalities similar to those observed in dyslexic brains were present in 3 out of the 10 non-dyslexic samples investigated, but appeared in significantly smaller numbers (1-2 per brain) and located away from language-related areas, predominantly in the cingulate cortex (Kaufmann & Galaburda, 1989).”

With reference to the possibility of including percentages of abnormalities, unfortunately the data presented in the original articles is very limited and does not allow for robust quantitative analyses of this sort, which we agree would be helpful

6. In Fig.3, the indicated dashed line according to the legend is a solid line.

Response: Thank you very much for bringing this to our attention. We have rectified this and redrawn the figure with the dashed line.

7. Page 11 line 50, referring to other neurodevelopmental disorders such as ASD and schizophrenia, there are much more recent databases and publications instead of the Xu et al., 2012 paper.

Response: We have now also included in this sentence in the 2^nd^ paragraph of section 4.2, now in p. 21, the latest data from the SFARI database from April 2018 as follows: “…(Xu et al., 2012), and the latest update from April 2018 of the database SFARI Gene lists over 1000 candidate genes for autism, of which only 25 are considered as high-confidence candidates (Abrahams et al., 2013).” The last cited reference has been added to the Reference list.

**Reviewer 2**

Comments to the Author

I thoroughly enjoyed reading this extremely well-constructed, clearly articulated, and ultimately sobering review article. It could be published “as is” in my view, so the following comments are purely meant as potential things to be considered and are entirely of the minor variety.

-	Page 4, Line 27: Is the VWFA (poorly named – see e.g. Vogel, Petersen, and Schlaggar, 2014) really unilateral?

Response: The original manuscript pointed out a role for this brain region beyond reading, but this was still somewhat limited. We have edited the sentence originally in p. 4, lines 26-29 (and now at the start of p. 6) to capture the much broader function proposed for it as follows: “An area of the left fusiform gyrus known as the visual word form area responsible for word recognition is generally considered as an important part of the reading circuitry (Logothetis and Sheinberg 1996, Dehaene & Cohen 2011), although this cortical region appears to serve functions that go beyond reading (Cohen et al. 2002; Vogel et al. 2014)”. Our reference to its unilaterality is in accordance with the neurolinguistics literature (e.g. Dehaene & Cohen 2011 cited above). These modifications involved the addition of the last mentioned reference and that from Vogel et al. 2014, and the removal of Dehaene et al. 2005 from the References list.

-	One observation from our own work is that when considering connectivity models of reading, one might also need to consider what the task of learning to read involves – that is, the association of already learned phonological information with orthographic inputs that children only receive exposure to after language has already emerged. That is, the kids need to learn a large set of visual-auditory cross-sensory associations and this must necessarily involve communication between visual and auditory regions/representations (see Hahn, Foxe and Molholm, 2014). I realize this is not central to the arguments being made in this review and mention it here only as an aside. However, this observation goes to something I note in the paper/field as a whole, and that is that the actual human deficit is not often considered in detail when animal model work is being conducted. This also goes to the early days of this work when the neuronal migration thesis was first emerging. It is a puzzle why migratory issues were assumed to only affect reading but not the basic language functions of these areas.

Response: Thanks for the interesting comments. We very much share your view that animal models do not capture the human behaviour/deficit well – this is likely to be partly due to the need to focus on specific endophenotypes of human dyslexia which have parallel behaviours in animals. There is a danger of over simplification too in trying to compare processes which are intrinsically different, especially when looking at the effect of a specific gene. In our experimental work (e.g. our Guidi et al 2017 paper in Cerebral Cortex) we are very careful to avoid any direct comparisons between the defect in auditory processing we identified in some of the mutants under study, with the human phenotype. The other point, on the assumption that defects in migration would specifically affect reading, is also extremely pertinent, particularly given how comorbid these disorders generally are.

-	On page 11 (lines 28 and 29), I was struck by the concluding statement, which seems altogether too accommodating of the prior work. The authors have really made a quite convincing argument by this stage that such a link has little to no convincing evidence and may wish to strengthen this statement.

Response: We have altered this statement in section 4.1.3, now in p. 20, as follows: “It is possible that future evidence shows this relationship to hold but, on the face of the current evidence base, the putative link between these genes and neuronal migration lacks evidential support and can no longer be mentioned without a statement of the known inconsistencies”

-	Bottom of page 11: I would point the authors to Kevin Mitchell’s recent EJN editorial on the candidate gene approach, which seems highly germane to this section of the paper -- <https://onlinelibrary.wiley.com/doi/abs/10.1111/ejn.13801>

Response: Thank you for bringing Kevin Mitchell's editorial to our attention. It is important to recognise that there are big challenges that go well beyond the study of language and this piece highlights that very well. We have added a sentence to capture this at the end of the 2^nd^ paragraph in section 4.2, now in p.21, as follows: "Nonetheless, important challenges remain in neurogenomics more generally, not only in the study of language, as highlighted by Mitchell (2018)." The cited reference has been added to the Reference list.

Typographic:

Page 5, line 35: “neurons ectopias” needs to be corrected.

This text previously reading “…and misplaced neurons (ectopias)…”, at the beginning of section 3, now in p. 8, has been changed to “…and misplaced neurons (**i.e.** ectopias)…”

Page 6, line 1: Pluralize “dysgenesis” – “dysgeneses”.

Text at the beginning of 2^nd^ paragraph in section 3, now in p. 9, has been corrected as indicated

Page 6, Line 6: “method” should be “methods”.

This falls in a sentence that has been changed to address comment #5 from Reviewer 1

Page 7, line 41: I would suggest replacing “mirror in” with “closely align with”.

Text at the end of 4^th^ paragraph in section 4, now in p. 12, has been edited as suggested

Page 8, line 3: Give date of Google Scholar search.

Date “(November 2017)” added (now in p. 13)

Page 12, line 20: “lights” should be “light”.

Corrected (1^st^ line of 2^nd^ paragraph in p. 22)

Page 12, line 26: Remove “study” where it says “exome sequencing study”.

Corrected (7^th^ line of 2^nd^ paragraph in p. 22)

Page 14, line 5: Shouldn’t “Viewed from a … lens” be “Viewed through a … lens”.

Corrected (1^st^ line of last paragraph in p. 25)

Page 15, lines 26-27: “on the face” should probably read “in the face”.

Corrected as suggested (5^th^ line of section 5, p. 28)

Page 15, line 43 “efforts of uncovering” should probably read “efforts to uncover”.

Corrected as suggested (8^th^ line of 2^nd^ paragraph of section 5, now in p. 29)

Abstract, last line, additional change (not requested by reviewers):

Page 2, abstract “anatomical and genetic” replaced by “neurobiological”

Please note that these typographic corrections have been entered without any indication of text changed (boldering or underlining).
